# Plasma Surface Polymerized and Biomarker Conjugated Boron Nitride Nanoparticles for Cancer-Specific Therapy: Experimental and Theoretical Study

**DOI:** 10.3390/nano9121658

**Published:** 2019-11-21

**Authors:** Elizaveta S. Permyakova, Liubov Yu. Antipina, Philipp V. Kiryukhantsev-Korneev, Andrey M. Kovalskii, Josef Polčak, Anton Manakhov, Kristina Yu. Gudz, Pavel B. Sorokin, Dmitry V. Shtansky

**Affiliations:** 1 National University of Science and Technology “MISIS”, Leninsky prospect 4, 119049 Moscow, Russia; permyakova.elizaveta@gmail.com (E.S.P.); antipinalyu@gmail.com (L.Y.A.); kiruhancev-korneev@yandex.ru (P.V.K.-K.); andreykovalskii@gmail.com (A.M.K.); ant-manahov@yandex.ru (A.M.); kristinkagudz@mail.ru (K.Y.G.); pbsorokin@gmail.com (P.B.S.); 2 Laboratory of New Materials Simulation, FSBI Technological Institute for Superhard and Novel Carbon Materials, 7a Tsentralnaya street, Troitsk, 108840 Moscow, Russia; 3 CEITEC-Central European Institute of Technology, Brno University of Technology, Technická 3058/10, 61600 Brno, Czech Republic; polcak@fme.vutbr.cz; 4 Institute of Physical Engineering, Brno University of Technology, Technicka 2896/2, 61669 Brno, Czech Republic

**Keywords:** BN nanoparticles, chemical vapor deposition, plasma surface polymerization, folic acid conjugates, drug delivery nanocarriers, density functional theory

## Abstract

A new low-pressure plasma-based approach to activate the surface of BN nanoparticles (BNNPs) in order to facilitate the attachment of folate acid (FA) molecules for cancer-specific therapy is described. Plasma treatment of BNNPs (BNNPs^PT^) was performed in a radiofrequency plasma reactor using ethylene and carbon dioxide monomers. The carboxyl groups deposited on the surface of BNNPs^PT^ were activated by N,N’-dicyclohexylcarbodiimide (DCC) and participated in the condensation reaction with ethylene diamine (EDA) to form a thin amino-containing layer (EDA-BNNP^PT^). Then, the DCC-activated FA was covalently bonded with BNNPs^PT^ by a chemical reaction between amino groups of EDA-BNNPs^PT^ and carboxyl groups of FA. Density functional theory calculations showed that the pre-activation of FA by DCC is required for grafting of the FA to the EDA-BNNPs^PT^. It was also demonstrated that after FA immobilization, the electronic characteristics of the pteridine ring remain unchanged, indicating that the targeting properties of the FA/EDA-BNNPs^PT^ nanohybrids are preserved.

## 1. Introduction

The current therapies are not sufficient to provide effective treatment of different forms of cancers. Targeted drug delivery (TDD) has become a widely used cancer research strategy to solve the main problems related to adverse effects of chemotherapeutic agents on healthy cells [1]. An important additional advantage of the TDD approach is the ability to create therapeutic systems with prolonged action [2]. Due to modern nanoindustry achievements, various types of nanoparticles (NPs) were synthesized and utilized as promising nanocarriers for cancer therapy. Grafting particular biomarkers that are overexpressed specifically on tumor cells enables the targeted delivery of therapeutical agents, thereby minimizing toxic side effects for the whole body. However, to develop therapeutically effective and biologically safe vehicles for TDD it is necessary to select a targeting molecule, a therapeutic agent, and a promising chemically stable and biologically safe support and then find a way to combine them into a single system [3,4].

Plasma surface functionalization and polymerization are powerful tools for NP surface activation, since the modification process can be well controlled and eco-friendly [5]. Plasma treatment allows the deposition of ultrathin plasma polymer using organic monomers that do not polymerize under conventional chemical conditions. In terms of gas pressure, all plasma processes can be divided into two main groups: low-pressure (LP) plasma modification and non-thermal atmospheric-plasma (AP) treatment [6]. Table 1 compares the stability of functional groups formed at LP and AP plasma treatments.

Oxygen-plasma treatment is frequently used to remove carbon contamination from the NP surfaces. The conversion of hydrocarbons into volatile compounds, such as CO, CO_2_, and H_2_O, allows one to remove them by pumping and to create the appropriate conditions for enhanced adhesion between the deposited polymer film and the NP surface [18]. In order to produce plasma polymer film containing high concentrations of amino functional groups, plasma polymerization of cyclopropylamine (CPA) was performed. The monomer selection was carried out based on available literature data, which have shown high stability and reactivity of the CPA films. Recently, LP plasma polymerization of CPA to functionalize ZnO, Al_2_O_3_, and ZrO_2_ NPs has been successfully tested [19]. TEM images clearly demonstrated that a thin (5 nm) plasma polymer film was formed on the surface of individual NPs, while the remaining NPs remained uncoated.

Both LP and AP plasmas were used to activate the polyethylene surfaces [20]. The influence of plasma process parameters, such as operating frequencies (40 kHz and 13.56 MHz at LP) and dielectric barrier discharge (50 Hz at AP), on the polymer surface activation were compared. A significant increase in surface energy and improved wettability was observed after LP plasma processing at 40 kHz, whereas AP plasma treatment was less effective toward surface activation at the same power couplings. Comparing the two methods, it can be noted that the absence of a vacuum system is the main advantage of the non-thermal AP plasma treatment, whereas LP plasma processing usually leads to the formation of more stable films containing a higher amount of functional groups on their surface.

Due to high chemical stability [21], different inorganic NPs, such as mesoporous silica [22], iron oxide [23], hexagonal boron nitride (h-BN) [24], and carbon nanotubes [25], have been developed and tested in NP-based cancer therapy. Among them, h-BN nanoparticles (BNNPs) are of particular interest due to their excellent biocompatibility [26], high drug loading capacity [27], and therapeutic efficacy toward tumor cells with multiple drug resistance [28].

Silver NPs were precipitated on the surface of BNNPs to provide coupling of folic acid (FA) with BNNPs [29]. The shortcomings of this strategy include difficulties in obtaining a uniform Ag NP size and their uniform distribution on the carrier surface. As a result, the FA-coated surface area was relatively small. Herein, we describe a new LP plasma-based approach to activate the surface of BNNPs to facilitate the attachment of folates, which are recognized as biomarkers for targeted chemotherapeutic drug delivery to cancer cells [30,31]. BNNPs were obtained by a chemical vapor deposition (CVD) process (Step (i)). Carboxy-containing plasma polymer was deposited on the surface of BNNPs (hereafter referred to as BNNPs^PT^) by means of a radiofrequency plasma reactor using ethylene and carbon dioxide monomers (Step (ii)). FA molecules were then conjugated to BNNPs in three consecutive stages (labeled as Steps (iii)–(v)): (iii) FA preactivation by N,N′-dicyclohexylcarbodiimide (DCC); (iv) NH_2_-functionalization of polymer-coated BNNPs with ethylenediamine, EDA (hereafter designated as EDA-BNNPs^PT^); and (v) final conjugation of DCC-activated FA to modified BNNPs (hereafter abbreviated as FA/EDA-BNNPs^PT^). Density functional theory (DFT) calculations were carried out to uncover the FA/EDA-BNNPs^PT^ chemical bonding mechanism. For that, a detailed energetic analysis of the atomic structure and stability of the FA/EDA-BNNPs^PT^ system was performed.

## 2. Materials and Methods

### 2.1. Materials

The following reagents were used: FA and EDA (PanReac AppliChem, Darmstadt, Germany); DCC and dimethyl sulfoxide, DMSO (Prime Chemical Group, Moscow, Russia); boron powder (AVIABOR, Dzerzhinsk, Russia); ammonia solution, dichloromethane, formic acid, and acetonitrile (Cupavnareactive).

### 2.2. Synthesis of BNNPs

BNNPs were synthesized in a boron oxide CVD process using a vertical induction-heating furnace, operating at 41.4 kHz [28]. The furnace consisted of a quartz cylindrical chamber, graphite susceptor, BN-based ceramic reactor, gas supply, and exhaust gas systems. The BN crucible with a precursor was placed at the bottom of reactor above the argon inlet. Precursor powder mixture of B (>99%), MgO (analytical grade), annealed in air at 450 °C for 1 h, and FeO (pure) taken in a molar ratio of 3.5:0.1:1 was used as a source of boron oxide vapor. Synthesis was carried out under the temperature gradient along the height of reactor from 1430 °C in the precursor zone to 700–750 °C in the BNNPs outlet zone. The ammonia and argon gas flows were controlled at 100 and 500 cm^3^/min, respectively. After the synthesis for 8 h, a thick white-colored deposit was observed in the collecting crucible and on the inner surface of the reactor.

### 2.3. Plasma Surface Polymerization of BNNPs

The deposition of carboxy-containing plasma polymer was carried out using a UVN-2M vacuum system evacuated to a pressure below 5 × 10^−3^ Pa. The capacitively coupled radio-frequency (RF) plasma was generated by a Cito1310-ACNA-N37A-FF (Comet) RF power supply unit coupled with a RFPG-128 plasma generator (Beams&Plasmas). High-frequency power (500 W, 13.56 MHz) was supplied in a pulsed mode (duty cycle 5%, pulse duration 2 ms). The deposition time was 10 min. Ar (99.998%), CO_2_ (99.995%), and C_2_H_4_ (99.95%) gases were used as precursors. The gas flow rates were set at 0.4, 2.5, and 3.5 sccm for C_2_H_4_, CO_2_, and Ar, respectively. Gas flow was controlled using a Multi Gas Controller 647C (MKS). Working and residual gas pressures were measured by a VMB-14 unit (Tokamak Company) and a D395-90-000 BOC Edwards controller. BNNP suspension in isopropyl alcohol (4 mg/mL) was sonicated for 10 min. Then, 30 mL of the BNNP suspension was applied to the glass surface and dried. The distance between the RF-electrode and the substrate was 8 cm. After deposition of the carboxy-containing polymer, the BNNPs were washed off the glass substrate for structural characterization. To analyze the elemental composition and thickness of plasma polymer, the plasma modified BNNPs were characterized by Fourier-transform infrared (FTIR) spectroscopy and X-ray photoelectron spectroscopy (XPS).

### 2.4. NH_2_-Functionalization of Carboxy-Modified BNNPs

NH_2_-functionalization of the BNNPs^PT^ was carried out according to the method described elsewhere [32]. Briefly, 40 mg of BNNPs^PT^ was added into 10 mL of DMSO after which the mixture was dispersed for 15 min. Then, 5 mg of DCC was added to the solution and stirred at room temperature for 10 min. Finally, 10 μL of amino-containing compound (EDA) was added to the reaction mixture and stirred at room temperature for 2 h. The particles were rinsed three times in distilled water and dried. The NH_2_-functionalized BNNPs (EDA-BNNPs^PT^) were characterized by XPS and FTIR spectroscopy.

### 2.5. Conjugation of FA to EDA-BNNPs^PT^

Ten milligrams of FA were dissolved in 10 mL of DMSO and then 2 mg of DCC was added. The mixture was added to a pre-dispersed solution of EDA-BNNPs^PT^ in 10 mL of methylene chloride (CH_2_Cl_2_). The reaction mixture was stirred at room temperature for 24 h, then the FA/EDA-BNNPs^PT^ were rinsed in distilled water and dried. The FA/EDA-BNNPs^PT^ powder samples were characterized by XPS, FTIR spectroscopy, and Fourier-transform ion cyclotron resonance mass spectrometry (FT ICR MS).

### 2.6. Material Characterization

The as-synthesized BNNPs and their conjugates were characterized using a scanning electron microscope JSM-7600F (JEOL) equipped with the energy-dispersive X-ray (EDX) detector and a transmission electron microscope JEM 2100 (JEOL). Chemical and phase compositions were studied by means of EDX spectroscopy using an 80 mm^2^ X-Max EDX detector (Oxford Instruments) and FTIR spectroscopy with a Vertex 70v vacuum spectrometer (Bruker) in the range 400−4000 cm^−1^. XPS spectra of the BNNPs, BNNPs^PT^, EDA-BNNPs^PT^, and FA/EDA-BNNPs^PT^ samples were recorded on an Axis Supra instrument (Kratos Analytical Ltd., Manchester, UK) equipped with a monochromatic Al K_α_ X-ray source (hυ = 1486.6 eV). The pass energy and X-ray beam current were set to 40 eV and 15 mA, respectively. The acquired spectra were fitted using CasaXPS software as described elsewhere [16,17]. The structure of FA/EDA-BNNPs^PT^ was additionally studied by FT ICR MS. To determine the molecular formula and ion type, ChemCalc software was used [33]. The FA/EDA-BNNPs^PT^ nanohybrids were added into the mixture of water−acetonitrile (50/50) with 0.1% formic acid and then centrifuged. The supernatants were analyzed by FT ICR MS using an Apex Ultra instrument (Bruker) with electrospray ionization at ionizing potential on a capillary of 4 kV. Zeta potentials were measured using a Zetasizer Nano ZS system (Malvern Instruments).

### 2.7. Atomistic Simulations

The atomic structure and stability of the FA conjugated to BNNP were analyzed using density functional theory (DFT) [34,35]. The generalized gradient approximation (GGA) using the normalized Troullier–Martins pseudopotentials [36] in the SIESTA software package was applied [37,38,39]. As a basis for atomic localized orbitals, numerical pseudoatomic wave functions were used. To neglect intermolecular interactions, the system was modelled as a supercell with the sufficiently large space between slabs (≥15 Å). The geometry of structures was optimized until residual forces became less than 0.03 eV/Å. The real-space mesh cutoff was set to at least 175 Ry. The Monkhorst–Pack [40] special *k*-point scheme was used with *k*-grid cutoff equaled to 6 and 24 Å for geometry relaxation and electronic structure calculations, respectively.

## 3. Results and Discussion

### 3.1. Preparation and SEM Characterization of FA-BNNPs Conjugates

**Figure 1:**
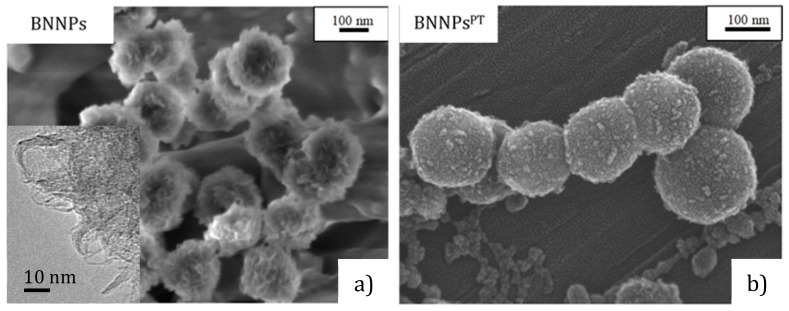
SEM (**a**,**b**) and TEM (inset in (**a**)) images of BN nanoparticles (BNNPs) and their conjugates.

Microstructures of as-synthesized and plasma polymerized BNNPs are depicted in Figure 1. As-fabricated BNNPs have an almost spherical shape and size of 150−250 nm. The BNNP surface is formed by numerous h-BN nanosheets, as shown in Figure 1a (inset). After the plasma treatment, the BNNPs^PT^ were covered by a thin layer of plasma polymer that significantly changes the BNNP morphology. The diameter of BNNPs^PT^ slightly increased to 170–270 nm, thereby suggesting that the thickness of the polymer layer is about 10 nm.

**Figure 2:**
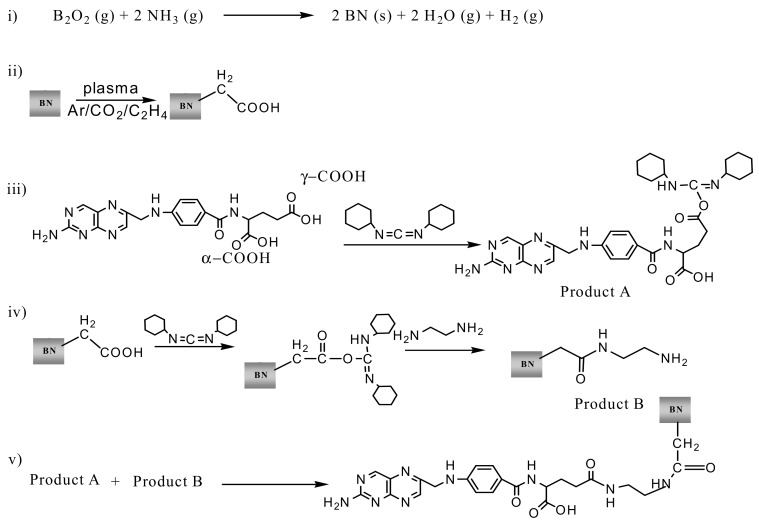
Scheme of BNNP fabrication and subsequent modification: BNNP synthesis using the reaction of boron oxide vapor with ammonia (**i**), plasma polymerization (**ii**), folate acid (FA) activation using DCC (**iii**), fabrication of EDA-BNNPs^PT^ (**iv**), and FA/EDA-BNNPs^PT^ (**v**) complexes.

Figure 2 illustrates five main stages of the FA/EDA-BNNP^PT^ conjugate fabrication: (i) BNNP synthesis, (ii) their plasma polymerization, (iii) FA activation, (iv) fabrication of EDA-BNNPs^PT^, and (v) conjugation of the pre-activated FA to the EDA-BNNPs^PT^, as well as the chemical structures of the modified BNNPs. The carboxyl groups deposited on the surface of BNNPs by plasma treatment (ii) are activated by DCC and participate in the condensation reaction with EDA to form a thin amino-containing layer on the BNNP surface (iv). Then, the carboxyl-groups of FA were preactivated by DCC (iii) and the FA was grafted to the surface of BNNPs^PT^ through chemical interaction between amino groups of EDA-BNNPs^PT^ and carboxyl groups of DCC-activated FA (v).

### 3.2. FTIR Spectroscopy 

**Figure 3:**
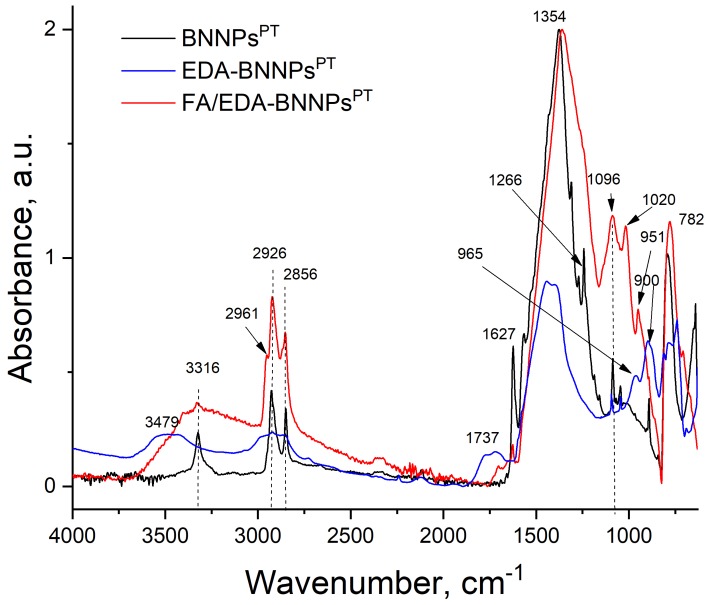
FTIR spectra of functionalized BNNPs.

The FTIR spectra of the BNNPs^PT^, EDA-BNNPs^PT^, and FA/EDA-BNNPs^PT^ nanohybrids are presented in Figure 3. The BNNPs sample [27] (not shown) reveals a strong asymmetric band at approximately 1354 cm^−1^ resulting from the B−N stretching vibration, and a less intense band at 772 cm^−1^ associated with the B−N−B bending vibration [41]. The FTIR spectrum of CO_2_/C_2_H_4_ plasma polymer (BNNPs^PT^ spectrum in Figure 3) shows several broad peaks located in the ranges of 3590–3340 (-OH groups), 3030–2780 (hydrocarbon), and 1820–1650 cm^−1^ (carboxyl/ester groups). In addition, few peaks observed at 1096, 965, and 900 cm^−1^ were assigned to C-C-(O)-C (esters), C-OH stretching, and C=C- bending vibrations, respectively. After NH_2_-functionalization of BNNPs^PT^ with EDA, new sharp peaks at 3316, 1627, and 1266 cm^−1^ appeared, which were assigned to the amino groups, C=O amide, and C-N stretch molecular motion, respectively. After the grafting of FA, the intensity of peak at 1096 cm^−1^ (C-C-(O)-C groups) noticeably increased and additional peaks at 2961 cm^−1^ (C-H_3_) and 1020 cm^−1^ (C-OH stretching) were observed. The pronounced peak at 1096 cm^−1^ can be explained by the contribution of C-C bending from folic acid [42,43,44]. To obtain an activated FA derivative (product B), carbodiimide was added to folic acid (stage 3 in Figure 2). Since the FA contains an amino group, polymerization of FA may occur due to the interaction of the activated carboxyl group with the free amino group resulting in the formation of a peptide bond. Thus, not a single folate molecule is attached, but a whole chain, which leads to an increase in the folate peak intensities, and, in particular, the C-C stretching band.

### 3.3. XPS Spectroscopy

**Figure 4:**
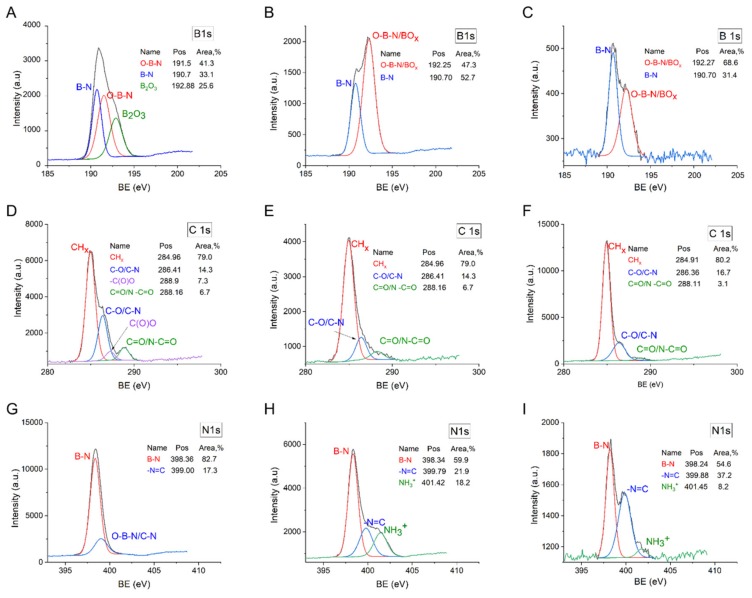
High-resolution XPS B 1s (**A**–**C**), C 1s (**D**–**F**), and N 1s (**G**–**I**) spectra of BNNPs^PT^ (**A,D,G**), EDA-BNNPs^PT^ (**B,E,H**), and FA/EDA-BNNPs^PT^ (**C,F,I**) samples. BE: binding energy.

Elemental composition and chemical bonds of the BNNPs^PT^, EDA-BNNPs^PT^, and FA/EDA-BNNPs^PT^ nanohybrids were studied by XPS. High-resolution XPS B 1s, C 1s, and N 1s spectra are illustrated in Figure 4. The XPS B 1s spectra of the EDA-BNNPs^PT^ and FA/EDA-BNNPs^PT^ samples were deconvoluted into two peaks located at 190.7 and 192.9 eV, corresponding to B-N and B_2_O_3_, respectively. Moreover, the XPS B 1s spectrum of the BNNPs^PT^ sample had an additional peak located at 191.5 eV, corresponding to the O-B-N bonds. The high-resolution XPS C 1s spectrum of the BNNPs^PT^ sample was deconvoluted into four peaks positioned at 285.0, 286.4, 288.1, and 288.9 eV, which could be attributed to CH_x_, C-O/C −N, C=O/N-C=O-, and C(O)O, respectively. The C(O)O component was not observed after further NH_2_-functionalization of BNNPs^PT^ with EDA and final conjugation of FA to the EDA-BNNPs^PT^ surface. The XPS N 1s spectrum of the BNNPs^PT^ was resolved into two main components attributed to B-N (398.3 eV) and O-B-N/C-N (398.9 eV). In the XPS N 1s spectrum of the EDA-BNNPs^PT^ sample, additional features can be seen at 399.6 and 401.8 eV due to the contribution from amide N-C=O bonds and protonated free amino groups appearing from EDA. In the XPS N 1s spectrum of the FA-BNNPs sample, the N-C=O peak shifts to lower binding energy (BE) at 399.8 eV and gained its intensity. On the contrary, the intensity of the NH_3_^+^ peak was observed to significantly decrease. This indicates the contribution from C=N-moieties typical for the folate structure. Thus, the XPS results clearly demonstrate the chemical bond of the FA molecules with the EDA-BNNPs^PT^ surface through amide linkage.

The grafting of EDA and FA to the surface of BNNPs^PT^ was quantitatively analyzed by calculating the N/B ratio. The N/B value in the BNNPs^PT^ determined from their XPS spectra is equal to 0.77, as shown in Table 2. The N/B ratio increased to 0.9 after BNNP surface fictionalization and further to 1.01 after subsequent FA cross-linkage. This indicates grafting ethylenediamine (EDA-BNNPs^PT^) and folic acid (FA/EDA-BNNPs^PT^) molecules. The additional increase in nitrogen content after EDA and FA modifications was calculated according to the following equations.

(1) $$\Delta N_{EDA-BNNPs}{}^{PT}=\left[\frac{N_{EDA-BNNPs}{}^{PT}}{B_{EDA-BNNPs}{}^{PT}}-\frac{N_{BNNPs}{}^{PT}}{B_{BNNPs}{}^{PT}}\right]\times {\left[B\right]}_{EDA-BNNPs}^{}{}^{PT}$$

(2) $$\Delta N_{FA/EDA-BNNPs}{}^{PT}=\left[\frac{N_{FA/EDA-BNNPs}{}^{PT}}{B_{FA/EDA-BNNPs}{}^{PT}}-\frac{N_{EDA-BNNPs}{}^{PT}}{B_{EDA-BNNPs}{}^{PT}}\right]\times {\left[B\right]}_{FA/EDA-BNNPs}^{}{}^{PT}$$

The obtained ΔN values are shown in Table 2. The total atomic concentration of C, O, and N atoms in the FA molecule grafted to the BNNPs (hereafter denoted as C_FA_) was estimated assuming that there was no hydrogen in the BNNPs (according to XPS data) and the FA has the chemical formula C_19_H_19_N_7_O_6_. Taking into account the number of N atoms in one FA molecule (C_19_H_19_N_7_O_6_), the C_FA_ = 3.8 at.% value was obtained by dividing the ΔN by 7 and multiplying this value by the total number of C (19), O (6), and N (7) atoms in one FA molecule. Note that additional C 1s and O 1s signals from both BNNP surface contaminations and BNNP-supported carbon tape can affect the results of quantitative XPS analysis. Thus, the obtained C_FA_ values were normalized to the total concentration of B and N atoms in BNNPs assuming equal contents of B and N in BN (denoted as C_BN_) using the following equation: C_FA_/C_BN_ = 3.8/7.5 = 0.51, where the C_BN_ value was determined as C_BN_ = 2[B] = 7.5. The obtained result indicates that FA molecules occupy approximately 51% of the BN surface. Thus, our new LP plasma-based approach provides a larger surface area covered with FA molecules compared with BNNPs decorated with Ag NPs [29].

### 3.4. Zeta Potential

**Figure 5:**
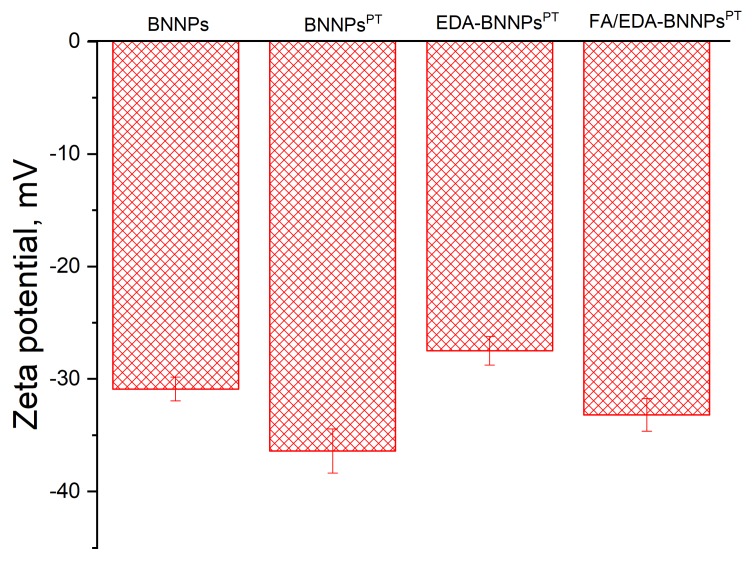
Zeta potentials of pristine and surface-functionalized BNNPs.

As-synthesized BNNPs have a negative surface charge (−30 mV) allowing the formation of stable suspensions, as shown in Figure 5. After plasma polymerization, the negative surface charge of the BNNPs^PT^ containing negatively charged carboxy-groups was observed to increase to −37 mV. Positively charged amino groups of EDA increased the zeta potential value of the EDA-BNNPs^PT^ conjugates up to −27 mV. A further shift of the surface charge towards a more negative value (−32.6 eV) indicates that the FA was successfully grafted to the EDA-BNNPs^PT^ conjugates.

### 3.5. Fourier-Transform Ion Cyclotron Resonance Mass Spectrometry

**Figure 6:**
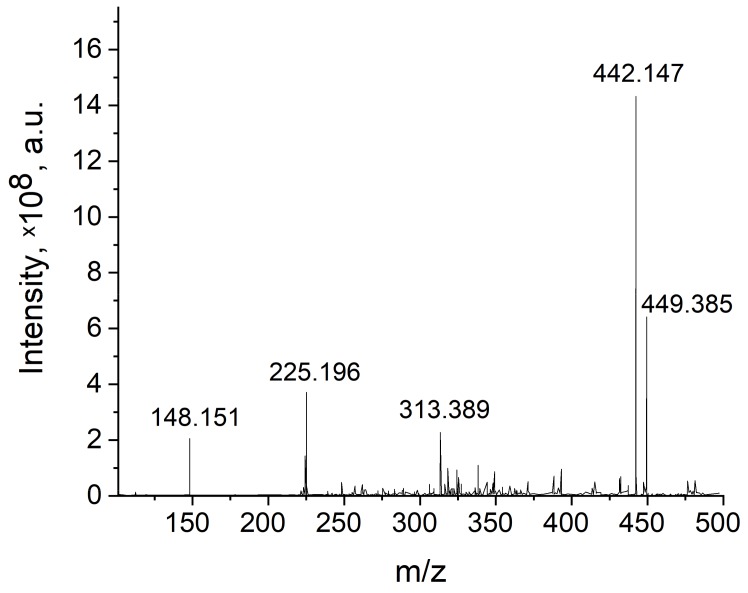
Fourier-transform ion cyclotron resonance mass spectrometry (FT ICR MS) spectrum of formic acid treated FA/EDA-BNNPs^PT^ sample.

The grafting of FA to the EDA-BNNPs^PT^ nanoparticles was additionally studied by a FT ICR MS method. Since BN nanocarriers are too heavy for FT ICR MS analysis, the FA/EDA-BNNPs^PT^ sample was first treated with formic acid to cleave the peptide bonds in the conjugates, after which the decomposed products were analyzed by FT ICR MS, as shown in Figure 6. Experimentally obtained mass to charge (m/z) values (right column in Table 3) were used to determine the main molecular ion types using a chemical Web service [33]. Besides peaks from the side product of N,N′-dicyclohexylurea, the characteristic FA peaks observed at 442.147 and 313.389 m/z and the peak at 148.151 m/z assigned to the glutamic acid part of FA clearly indicate that the FA was successfully conjugated to the BNNP surface.

### 3.6. Simulation of FA/EDA-BNNPs^PT^ Nanohybrids

To understand how the FA grafts to the EDA-BNNPs^PT^ conjugates, detailed theoretical analysis was performed using atomistic simulations by considering interaction of the FA containing various carboxyl groups with the BN surface. The experimental data indicate that the BNNP has a size of more than 10^2^ nm, which allows us to accept the assumption that its surface is flat. Since the electron transfer between the h-BN layers is almost absent, only a few atomic planes in the h-BN sheet were considered. This allows us to apply periodic boundary conditions and to consider a relatively small supercell in the calculations. The binding energies (BEs) were calculated during activation of FA with DCC, as well as at each step of BNNPs^PT^ modification with DCC, EDA, and DCC-activated FA. 

**Figure 7:**
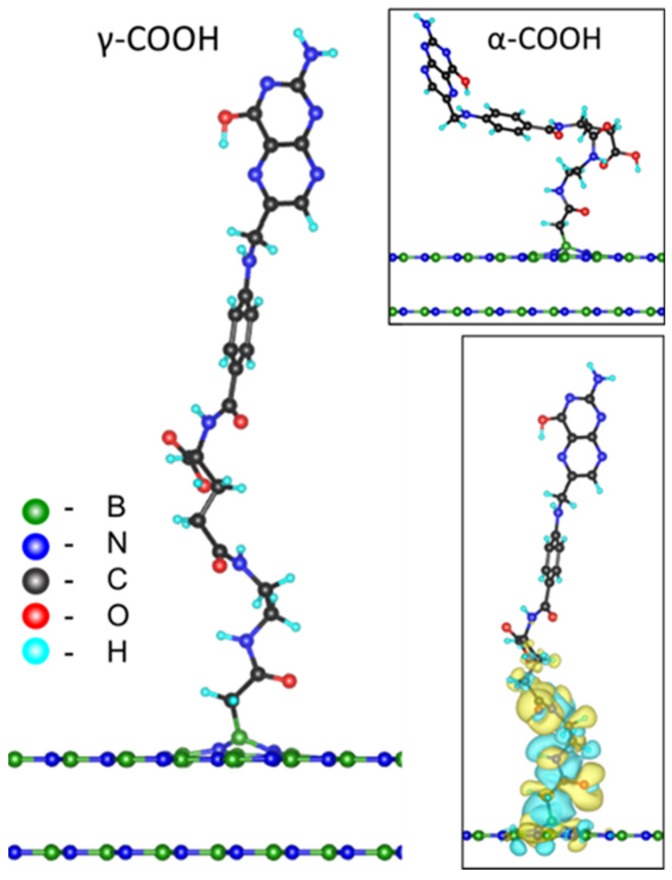
Schematics of FA/EDA-BNNPs^PT^ (γ-COOH). Top inset: another possible but energetically unfavorable type of bonding between EDA-BNNPs^PT^ and carboxyl groups of FA (α-COOH). Bottom inset: distribution of spatial charge density difference between FA/EDA-BNNPs^PT^ (γ-COOH) and corresponding freestanding parts. The loss and gain of charge are denoted by yellowish and bluish colors, respectively. The boron, nitrogen, carbon, oxygen, and hydrogen atoms are marked by green, blue, gray, red, and cyan colors, respectively.

During plasma surface polymerization, the –CH_2_-COOH groups were deposited on the BNNP surface. The high resistivity of the B–N π system to adsorption leads to remarkable chemical stability of the perfect h-BN surface, therefore carbon groups will mostly bind with surface topological defects, such as B or N vacancies. The BNNPs^PT^ containing COOH groups were NH_2_-functionalized with EDA (NH_2_-CH_2_-CH_2_-NH_2_) as shown in Figure 7. The EDA-BNNPs^PT^ system was further activated by DCC to immobilize FA.

At each step of BNNP modification, the BEs were calculated as the difference between system total energy with an absorbed molecule ($E_{tot}^{n}$) and each freestanding constituent part ($E_{m}^{}$, where *m* is FA, EDA, DCC-BNNPs^PT^, or BNNPs^PT^):

(3) $$E_{b}^{n}=E_{tot}^{n}-\sum E_{m}.$$

The calculated BE values are summarized in Table 4. Although the boron vacancy is energetically more favorable than the nitrogen one (the energy difference is ~1 eV), the BE of the carbon group -CH_2_-COOH to boron (in the case of nitrogen vacancy) is 0.5 eV lower than to nitrogen (in the case of boron vacancy). The interaction of inactivated FA with the EDA-BNNPs^PT^ sample is thermodynamically unfavorable (the BE of FA with EDA-BNNPs^PT^ (V_N_) through the γ-COOH group is positive and equal to 0.12 eV), whereas the use of DCC-activated FA reduces the BE to a negative value of ~−1.45 eV for the same system. The increase in binding energy (in absolute value) can be well explained by the formation of additional bonds during two processes—water formation from –H of the EDA amino group and –OH of the FA carboxyl group during FA activation, and conversion of DCC to dicyclohexylurea through an intermediate step of O-acylated dicyclohexylurea formation, as shown in Figure 2.

Since there are two carboxyl groups (marked as α- and γ-COOH groups in Figure 2) at the glutamic acid part of the FA, the BE of FA with EDA-BNNPs^PT^ hybrid may depend on the carboxyl group type. The calculation results obtained for the V_N_ show that the binding of FA to EDA-BNNPs^PT^ is more favorable through the γ-COOH group end of DCC-activated FA (BE = −4.32 eV (α-COOH) and BE = −5.09 eV (γ-COOH)), as shown in Table 4. Moreover, since the BE of DCC to FA is different (BE = −0.73 eV (α-COOH) and BE = −1.15 eV (γ-COOH)), the γ-COOH activation process is energetically more favorable compared to the α-COOH counterpart, as shown in Figure 2.

Preserving the electronic properties of the pteridine ring of FA unchanged is crucially important for further successful interaction of FA/EDA-BNNPs^PT^ nanoconjugate with folate receptors of cancer cells. To determine whether the FA electronic structure remains undamaged during the grafting of FA to EDA-BNNPs^PT^, the difference in spatially distributed electron density between the whole system and each constituent part was calculated as follows:

(4) $$\Delta \rho_{{\mathrm{FA}/\mathrm{EDA}-\mathrm{BNNPs}}^{\mathrm{PT}}}^{}=\rho_{{\mathrm{FA}/\mathrm{EDA}-\mathrm{BNNPs}}^{\mathrm{PT}}}^{}-\rho_{\mathrm{FA}}-\rho_{\mathrm{EDA}}-\rho_{{\mathrm{BNNPs}}^{\mathrm{PT}}}.$$

This allows us to understand the nature of the interaction between FA and EDA-BNNPs^PT^ because the charge redistribution due to the formation of covalent bonding may affect the electronic structure of the whole system. The distribution of spatial charge density difference $\Delta \rho_{{\mathrm{FA}/\mathrm{EDA}-\mathrm{BNNPs}}^{\mathrm{PT}}}^{}$ is illustrated in Figure 7 (bottom inset). It can be seen that the electronic density of the FA/EDA-BNNPs^PT^ nanohybrid is changed only in the region of FA-EDA bonding, whereas the electronic structure of the pteridine ring in FA is not disturbed with respect to the freestanding, unbound molecule. This result indicates that the properties of targeted molecules are not affected by grafting FA to the BNNPs^PT^ surface.

## 4. Conclusions

For the first time, a low-pressure plasma surface polymerization approach has been utilized to activate the surface of BN nanoparticles (BNNPs) in order to facilitate the immobilization of folate acid (FA) molecules for targeting folate receptors in cancer cells. The obtained results are summarized as follows.

1. The carboxyl groups deposited on the surface of BNNPs by plasma treatment (BNNPs^PT^) using ethylene and carbon dioxide monomers were activated by N,N′-dicyclohexylcarbodiimide (DCC) and participated in the condensation reaction with ethylene diamine (EDA) to form a thin amino-containing layer on the EDA-BNNP^PT^ surface.
2. FA molecules were successfully grafted to 51% of the BNNP surface through covalent bonding between amino groups of EDA-BNNPs^PT^ and carboxyl groups of DCC-activated FA.
3. Density functional theory calculations showed that the DCC pre-activation of FA is required for the formation of thermodynamically favorable bonding with EDA-BNNPs^PT^.
4. Grafting of FA to the EDA-BNNPs^PT^ does not affect the electronic structure of the pteridine ring, hereby indicating that the targeting properties of the FA/EDA-BNNPs^PT^ nanohybrids are preserved.

## Figures and Tables

**Table 1:** Stability of functional groups formed at low-pressure (LP) and atmospheric-plasma (AP) plasma treatments. XPS: X-ray photoelectron spectroscopy.

No.	Substrate	Gas	Groups	Plasma	N/C and O/C Ratio (XPS)	Stability, H (Thickness loss, %)	Ref
1	Si	Cyclopropylamine (CPA)	NH_2_	LP	0.24 (N/C)	20%, 48 h	[7]
2	Si	*n*-Heptylamine	NH_2_	LP	0.12 (N/C)	15%, 24 h	[8]
3	Ti	Allylamine	NH_2_	LP	0.20 (N/C)	50%, 24 h	[9,10]
4	Ti	Air/H_2_O_2_/TEOS (Tetraethoxysilane)	OH	LP	0.58 (O/C)	100%, 12 h (in boiling toluene)	[11]
5	Polymer	Allylamine Acrylic Acid	NH_2_, COOH	LP	0.20 (N/C) 0.64 (O/C)	-	[12]
6	Polymer	O_2_	OH, COOH	AP	0.09 (O/C)	55%, 24 h	[13]
7	Polymer	Air	OH, COOH	AP	0.43 (O/C)	-	[14]
8	Polymer	Air	OH, COOH	AP	0.68 (O/C)	-	[15]
9	Si	CO_2/_C_2_H_4_/Ar	OH, COOH	LP	0.45 (O/C)	39%, 24 h (high stability of COOH groups)	[16]
10	Si	Maleic Anhydride Vinyltrimethoxysilane	OH, COOH	AP	0.20 (O/C)	75%, 20 h	[17]

**Table 2:** Results of XPS analysis.

Sample	Concentration, at %				N/B	ΔN
	B	C	N	O		
**BNNPs^PT^**	**33.3**	16.8	25.6	24.3	0.77	
EDA-BNNPs^PT^	21.5	34.2	16.8	27.5	0.78	0.27
FA/EDA-BNNPs^PT^	3.7	71.0	3.8	21.5	1.01	0.86

**Table 3:** Main ions detected by FT ICR MS.

Compound	Formula	Molecular Ion Type	m/z Theoretical	m/z Experimental
N,N′-dicyclohexylurea	C_13_H_24_N_2_O	[M_2_ + H]^+^	449.385	449.385
Folic acid	C_19_H_19_N_7_O_6_	[M + H]^+^	442.138	442.147
Part of FA	C_14_H_12_N_6_O_3_	[M + H]^+^	313.297	313.389
N,N′-dicyclohexylurea	C_13_H_24_N_2_O	[M + H]^+^	225.196	225.196
Glutamic acid	C_5_H_9_NO_4_	[M + H]^+^	148.146	148.151

**Table 4:** The BE values (eV) for each reaction step during the fabrication of FA/EDA-BNNPs^PT^ nanohybrids.

Sample	V_N_		V_B_	
	Type of Carboxyl Group of FA			
	α-COOH	γ-COOH	α-COOH	γ-COOH
Activation of FA by DCC (FA*)	−0.73	−1.15	−0.73	−1.15
DCC + BNNPs^PT^	−2.65	−2.41	−2.75	−2.55
EDA + DCC-BNNPs^PT^	−3.89	−3.64	−4.23	−3.98
FA* + EDA-BNNPs^PT^	−4.32	−5.09	−4.70	−5.07
